# Respiratory variation in the internal jugular vein does not predict fluid responsiveness in the prone position during adolescent idiopathic scoliosis surgery: a prospective cohort study

**DOI:** 10.1186/s12871-023-02313-8

**Published:** 2023-11-06

**Authors:** Mimi Wu, Zhao Dai, Ying Liang, Xiaojie Liu, Xu Zheng, Wei Zhang, Jinhua Bo

**Affiliations:** 1 Department of Anesthesiology, Nanjing Drum Tower Hospital, The Affiliated Hospital of Nanjing University Medical School, Nanjing, People’s Republic of China; 2 Department of Anesthesiology, The Affiliated Yantai Yuhuangding Hospital of Qingdao University, Yantai, People’s Republic of China

**Keywords:** Internal jugular vein, Pulse pressure variation, Fluid responsiveness, Adolescent idiopathic scoliosis, Posterior spinal fusion, Doppler ultrasound

## Abstract

**Background:**

Respiratory variation in the internal jugular vein (IJVV) has not shown promising results in predicting volume responsiveness in ventilated patients with low tidal volume (Vt) in prone position. We aimed to determine whether the baseline respiratory variation in the IJVV value measured by ultrasound might predict fluid responsiveness in patients with adolescent idiopathic scoliosis (AIS) undergoing posterior spinal fusion (PSF) with low Vt.

**Methods:**

According to the fluid responsiveness results, the included patients were divided into two groups: those who responded to volume expansion, denoted the responder group, and those who did not respond, denoted the non-responder group. The primary outcome was determination of the value of baseline IJVV in predicting fluid responsiveness (≥15% increases in stroke volume index (SVI) after 7 ml·kg^-1^ colloid administration) in patients with AIS undergoing PSF during low Vt ventilation. Secondary outcomes were estimation of the diagnostic performance of pulse pressure variation (PPV), stroke volume variation (SVV), and the combination of IJVV and PPV in predicting fluid responsiveness in this surgical setting. The ability of each parameter to predict fluid responsiveness was assessed using a receiver operating characteristic curve.

**Results:**

Fifty-six patients were included, 36 (64.29%) of whom were deemed fluid responsive. No significant difference in baseline IJVV was found between responders and non-responders (25.89% vs. 23.66%, *p* = 0.73), and no correlation was detected between baseline IJVV and the increase in SVI after volume expansion (*r* = 0.14, *p* = 0.40). A baseline IJVV greater than 32.00%, SVV greater than 14.30%, PPV greater than 11.00%, and a combination of IJVV and PPV greater than 64.00% had utility in identifying fluid responsiveness, with a sensitivity of 33.33%, 77.78%, 55.56%, and 55.56%, respectively, and a specificity of 80.00%, 50.00%, 65.00%, and 65.00%, respectively. The area under the receiver operating characteristic curve for the baseline values of IJVV, SVV, PPV, and the combination of IJVV and PPV was 0.52 (95% CI, 0.38–0.65, *p*=0.83), 0.54 (95% CI, 0.40–0.67, *p*=0.67), 0.58 (95% CI, 0.45–0.71, *p*=0.31), and 0.57 (95% CI, 0.43–0.71, *p*=0.37), respectively.

**Conclusions:**

Ultrasonic-derived IJVV lacked accuracy in predicting fluid responsiveness in patients with AIS undergoing PSF during low Vt ventilation. In addition, the baseline values of PPV, SVV, and the combination of IJVV and PPV did not predict fluid responsiveness in this surgical setting.

**Trail registration:**

This trial was registered at www.chictr.org (ChiCTR2200064947) on 24/10/2022. All data were collected through chart review.

**Supplementary Information:**

The online version contains supplementary material available at 10.1186/s12871-023-02313-8.

## Introduction

Appropriate intraoperative fluid therapy aimed at optimizing cardiac output (CO) is important in decreasing postoperative complications and mortality, whereas inappropriate fluid management has been reported to be harmful [1–3]. Posterior spinal fusion (PSF) for the treatment of adolescent idiopathic scoliosis (AIS) has typically been associated with substantial fluid shifts, blood loss, and transfusion requirements [4, 5]. In this context, fluid administration is the first line therapy used to increase CO, and reverse hypovolemia and tissue hypoperfusion. However, fluid therapy increases CO in only half of patients when fluid responsiveness is not predicted [6, 7]. Therefore, the assessment of fluid responsiveness is essential to prevent fluid overload in patients who are fluid unresponsive.

Many predictors of fluid responsiveness have been investigated [8]. Traditional static parameters, such as central venous pressure (CVP) and pulmonary capillary wedge pressure, have been criticized for their lack of reliability in predicting the effects of fluid therapy [9, 10]. Subsequently, dynamic indicators based on heart-lung interactions, including pulse pressure variation (PPV) and stroke volume variation (SVV), have been widely used to predict preload responsiveness in mechanically ventilated patients [8, 11]. Unfortunately, these dynamic indices may have diminished reliability during lung-protective ventilatory (low tidal volume (Vt) ventilation or Vt ≤ 8 ml·kg^-1^) and prone position, which are commonly used during PSF [12, 13]. Furthermore, the measurement of these hemodynamic parameters requires invasive procedures and special monitoring equipment, thus further limiting their clinical application.

Recently, noninvasive ultrasound has been recommended to evaluate fluid status, because of its measurement reproducibility and ease of image acquisition. Among these ultrasound modalities [6, 14, 15], respiratory variations in the superior and inferior cava vena have been extensively studied in patients who are mechanically ventilated and spontaneously breathing [16–18]. Of note, measurements of the superior and inferior vena cava may fail to predict fluid responsiveness because of poor image quality caused by surgical drains, dressings, mediastinal air, morbid obesity or abdominal distension. Current evidence suggested internal jugular vein (IJVV) has shown promising results in predicting volume responsiveness in spontaneously breathing patients and ventilated patients with low Vt [19–22]. Additionally, unlike respiratory variation in the superior vena cava, measurement of the IJVV does not require transesophageal echocardiography, and sonographic visualization is much more easily accessible [23]. Although IJVV has been investigated in several studies, its accuracy in ventilated patients with low Vt in prone position has not been confirmed.

The primary aim of this study was to estimate whether IJVV, as assessed by Doppler ultrasound, might serve as a reliable predictor of fluid responsiveness in patients with AIS undergoing PSF during low Vt ventilation. Furthermore, we evaluated the ability of the baseline values of PPV, SVV, and the combination of IJVV and PVV to predict fluid responsiveness.

## Materials and methods

This prospective study was approved by the Research Ethics Committee of Nanjing Drum Tower Hospital affiliated with Nanjing University Medical School (No. 2022-287-02, dated 16 June 2022), and informed consent was obtained from each patient’s next of kin before surgery. This trial was registered at www.chictr.org (ChiCTR2200064947) on October 24, 2022. All data were collected through chart review.

### Patients

All patients between 12 and 18 years of age who were diagnosed with AIS, had a body mass index between 18 and 30 kg·m^-2^, and were scheduled for PSF were included. Their charts were screened for study eligibility. The exclusion criteria were as follows: cardiac dysfunction, arrhythmias, American Society of Anesthesiologists physical status ≥ III, internal jugular thrombosis, persistently poor quality arterial signal during the study, inability to obtain clear ultrasound images, contraindications to radial artery puncture, and known allergy to 6% hydroxyethyl starch (HES) 130/0.4.

### Anesthesia management

After arriving at the operating room, all patients underwent standard intraoperative monitoring of pulse oxygen saturation, noninvasive blood pressure, continuous electrocardiography, and heart rate (HR). Ringer’s solution (7 ml·kg^-1^·h^-1^) was administered before volume expansion. Anesthesia was induced with propofol (1 mg·kg^-1^), midazolam (0.04 mg·kg^-1^), and fentanyl (4 μg·kg^-1^), and neuromuscular block was achieved with intravenous vecuronium bromide (0.10 mg·kg^-1^). After endotracheal intubation, the patients were ventilated in volume-control mode with a Vt of 7 ml·kg^-1^ of the ideal body weight [24], an inspiratory to expiratory ratio (I:E) of 1:2, and a fraction of inspired oxygen of 0.60, without positive end-expiratory pressure. Moreover, the respiratory rate was set to maintain an end-tidal carbon dioxide concentration between 30 and 35 mmHg. All these respiratory parameters remained unchanged during the study period. Anesthesia was maintained with propofol (2–4 mg·kg^-1^·h^-1^), remifentanil (0.15 μg·kg^-1^·h^-1^), and cisatracurium (0.10 mg·kg^-1^·h^-1^), to maintain a bispectral index score of 40–60.

### Hemodynamic monitoring

After induction of anesthesia, a 20-G radial arterial line was inserted and connected to a pressure transducer. The invasive arterial blood pressure and PPV were continuously displayed on an Intellivue MP70 patient monitor (Philips Medical Systems, Germany) in real time. The radial arterial catheter was also connected to a dedicated transducer (FloTrac^TM^, Edwards Lifesciences, Irvine, CA, USA) to obtain the following hemodynamic parameters: SVV, stroke volume index (SVI), and cardiac index, which were displayed on a Vigileo^TM^ monitor (Edwards Lifesciences [4th generation algorithm]). Furthermore, CVP was obtained through an internal jugular vein (IJV) catheter. The pressure transducers were continuously adjusted to the level of the patients’ right atrium during the study.

Pulse pressure (PP) was defined as the difference between systolic and diastolic arterial pressures. The maximal pulse pressure (PPmax) and minimal pulse pressure (PPmin) values were determined during the same respiratory cycle. PPV was automatically computed with the following formula: PPV(%) = (PPmax-PPmin)/[( PPmax+PPmin)/2]. In addition, SVV was defined as the beat-to-beat SV variation from the mean value during the past 20 sec and calculated with the following equation: SVV=(SVmax –SVmin)/SVmean [25]. The mean values of PPV and SVV from three consecutive respiratory cycles were used for statistical analyses.

Sonographic measurement of IJVV was performed with a Philips CX50 ultrasound device (Philips Healthcare, Hamburg, Germany), equipped with a linear transducer. IJVV was measured by the same anesthesiologist with sufficient experience in ultrasound-guided IJV cannulation. The anesthesiologist performing the ultrasound examinations was not involved in this study and blinded to the hemodynamic parameters; a lead plate separated the monitor from the anesthesiologist and ultrasound device. The optimal short axis of the IJV was obtained at the level of the cricoid cartilage by placement of the transducer perpendicular to the skin on the patient’s neck in a transverse plane. The vein was identified with color Doppler imaging as well as by compression. Ultrasound measurements were performed on the left IJV, to minimize the risk of infection at the right IJV puncture site.

**Fig. 1:**
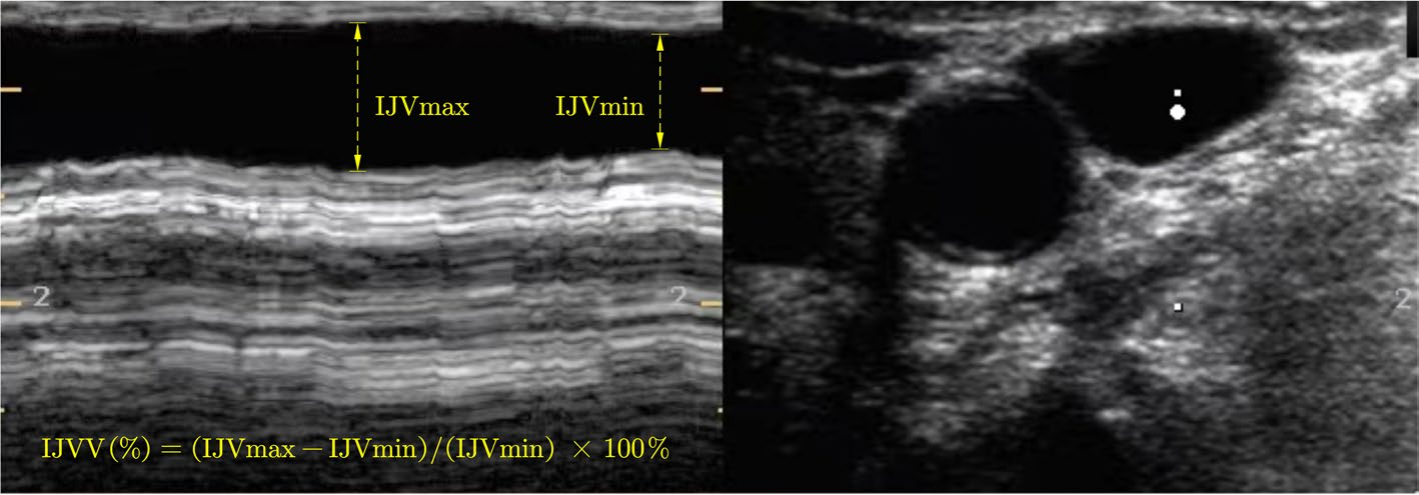
Representative ultrasound image of the IJVV. IJVV, respiratory variation in the internal jugular vein

An M-mode scan was used to record the IJV diameter at the end of inspiration (IJVmax) and expiration (IJVmin) over an entire respiratory cycle. The images were then frozen (Fig. 1). The IJVV was calculated with the following formula: IJVV (%) = (IJVmax − IJVmin)/(IJVmin) × 100%. The average values of IJVmax and IJVmin from three consecutive respiratory circles were used in the analysis.

### Study protocol

**Fig. 2:**
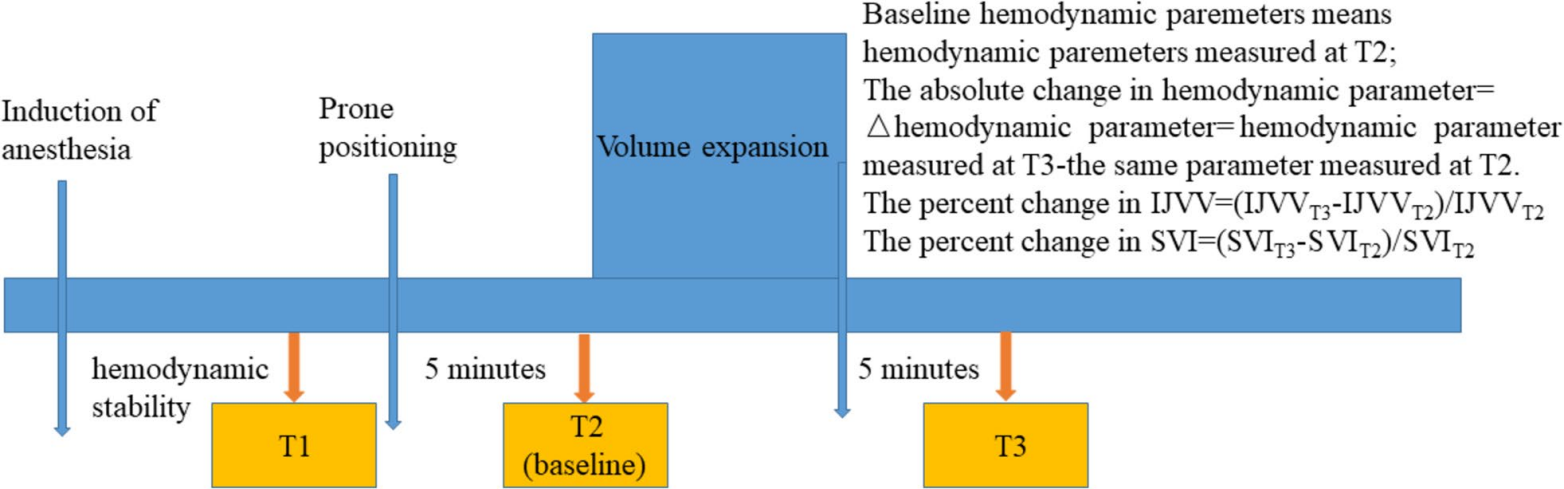
Study protocol and concepts of baseline hemodynamic parameters and the absolute and percentage changes in hemodynamic parameters. IJVV, respiratory variation in the internal jugular vein; SVI, stroke volume index

Three study time points were investigated. The first measurement started after induction of anesthesia, during a period of hemodynamic stability, defined as a change in invasive mean arterial pressure (MAP) of <10% within 5 minutes (T1) [26]. To ensure an absence of spontaneous breathing activity, the attending anesthesiologist monitored the curves displayed on the ventilator. After intravenous administration of propofol 1 mg·kg^-1^, patients were then turned to the prone position on six pads (two pelvic, two shoulder, and two chest supports) with the abdomen hanging free. Furthermore, their heads were placed straight down on soft pads, in line with their bodies. Five minutes after prone positioning, a second measurement was recorded (T2). Subsequently, volume expansion was performed by infusion of 6% HES 130/0.4 at 7 ml·kg^-1^ over 15 minutes. Five minutes after completion of intravenous fluid loading, the third measurement was recorded (T3) (Fig. 2). Patients were classified as responders if they had an increase in SVI ≥15% ((SVI_T3_-SVI_T2_)/SVI_T2_) after intravenous fluid loading between T2 and T3, and were otherwise classified as non-responders [27, 28]. The HR, MAP, CVP, PPV, SVV, SVI, cardiac index, IJVV, and dynamic lung compliance (Cdynamic = Vt/(peak inspiratory pressure − positive end-expiratory pressure)) were collected at each time point.

All these variables were measured without any external stimulation to the patient during this period, with a constant ventilator setting, in a hemodynamically steady state without use of inotropes or vasopressors. Furthermore, these parameters were recorded by an anesthesiologist who not involved in the study.

### Study endpoints

The primary outcome of this study was investigation of whether IJVV, as assessed by Doppler ultrasound, might be a reliable predictor of fluid responsiveness in patients with AIS undergoing PSF during low Vt ventilation. Secondary outcomes were estimation of the diagnostic performance of PPV, SVV, and the combination of IJVV and PPV in predicting fluid responsiveness in this surgical setting.

### Statistical analysis

Normality of the distribution of these variables was assessed with the Kolmogorov–Smirnov test. Normally distributed, continuous data are presented as mean ± standard deviation, whereas non-normally distributed data are presented as median and interquartile range. Categorical variables are presented as absolute numbers of patients (%). The effects of changes in volume expansion on hemodynamic and respiratory variables were assessed with paired t test for normally distributed data or Wilcoxon rank sum test for non-normally distributed data. Responder and non-responder groups were compared with independent t-test (if normally distributed) or Mann–Whiney U-test (if non-normally distributed). The hemodynamic and respiratory variables of all patients from T1–T3 were analyzed separately with repeated measures analysis of variance or a generalized linear mixed model test. *Post hoc* pairwise multiple comparisons analysis were performed using the Bonferroni correlation. Categorical data were analyzed with either χ2 test or Fisher’s exact test, as appropriate.

Spearman’s correlation coefficients or Pearson correlation tests were used to analyze the relationship between dynamic parameters and the increase in SVI after volume expansion. ROC curve analysis was used to assess the following variables potentially predicting fluid responsiveness: (1) IJVV, PPV, and SVV at T2, and (2) the combination of IJVV and PVV at T2. Comparisons of the areas under the ROC curves (AUCs) were conducted with the Delong method [29]. The optimal cutoff was determined by maximizing the Youden index (sensitivity + specificity − 1).

Because IJVV had not previously been studied in surgical patients in prone position with low Vt ventilation, we assumed an AUC for IJVV of at least 0.75, the minimum threshold necessary to consider a diagnostic test accurate [30]. Furthermore, to calculate the sample size of this study, we compared 0.75 and the null hypothesis (AUC = 0.50 and ratio in negative/positive groups of sample size = 1), then determined a sample size of 38 patients (with a two-sided type I error = 0.05, 1-β = 0.8) [26].

Statistical analyses were performed using SPSS27 (IBM, Armonk, NY, USA) and MedCalc (version 20.100; MedCalc Software Ltd., Ostend, Belgium). For all comparisons, a two-tailed *p* value < 0.05 was considered statistically significant.

## Results

### Study population

**Fig. 3:**
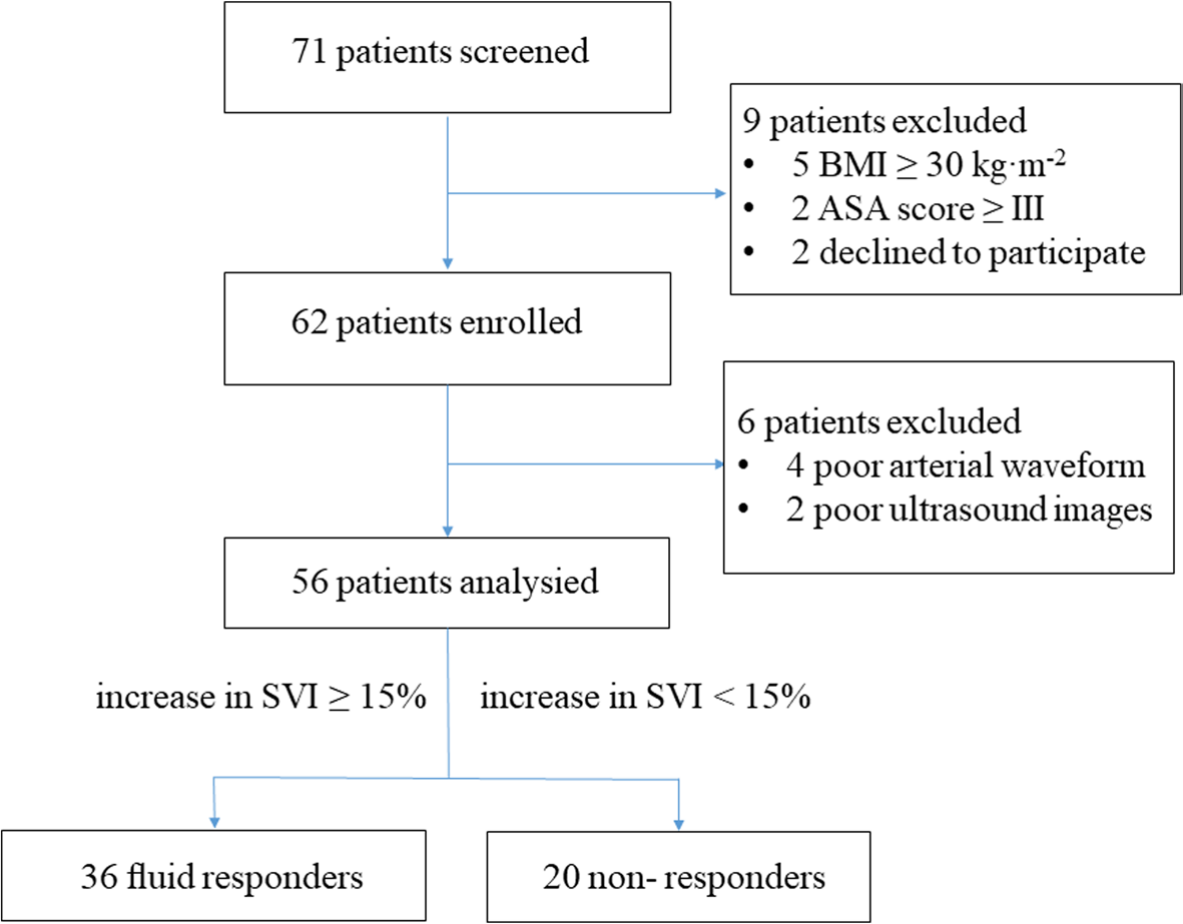
Flow chart of the study. BMI, Body mass index; ASA, American Society of Anesthesiologists; SVI, stroke volume index

A total of 71 patients diagnosed with AIS and scheduled for elective PSF surgery were initially identified. Nine patients were excluded because of body mass index ≥ 30 kg·m^-2^ (*n* = 5), American Society of Anesthesiologists physical status ≥ III (*n* = 2), or refusal to participate (*n* = 2). Thus, 62 patients were enrolled in the study. Six patients were not included because of poor arterial waveforms (*n* = 4) and poor ultrasound images (*n* = 2). Therefore, 56 patients were finally included in the analysis. Thirty-six patients (64.29%) were responders, and 20 patients (35.71%) were non-responders to 7 ml·kg^-1^ fluid loading (Fig. 3). The general characteristics of the patients are summarized in Table 1. No significant differences in baseline clinical or demographic characteristics were observed between responders and non-responders.

**Table 1:** General patient characteristics

Characteristics	Fluid responders (*n*=36)	Non-responders (*n*=20)	*P* value
Age (year)	14 [13–16]	15 [14–16]	0.19
Female, n (%)	24 (67%)	11 (55%)	0.39
Height (cm)	162 ± 11	159 ± 9	0.51
Weight (kg)	56.31 ± 9.81	49.95 ± 8.82	0.52
BMI (kg·m^-2^)	21.30 ± 2.61	19.68 ± 1.97	0.22
ASA score, I/II (n)	20/16	9/11	0.45
Hct before surgery (%)	35.25 ± 3.52	35.40 ± 4.16	0.86
Tidal volume (ml)	350.00 [350.00-423.75]	347.50 [300.00-375.00]	0.05
Volume administered after echo (ml)	647.85 ± 151.05	564.24 ± 100.99	0.68
Levels operated (n)	9 [7.25-11.00]	8 [6.25-9.75]	0.62
Cobb angle (°)	48.56 ± 4.99	47.10 ± 5.65	0.65
Surgery time (min)	182.08 ± 56.09	186.00 ± 62.88	0.35
Blood loss (ml)	600 [410-775]	700 [425-775]	0.76

Data are presented as the mean ± standard deviation, median [interquartile range], or the number of patients (%)

*BMI* Body mass index, *ASA* American Society of Anesthesiologists, *Hct* Hematocrit

### Changes in hemodynamic and respiratory variables among all patients over the course of the study

Prone positioning led to significant decreases in HR, MAP, IJVV, SVV, and PPV, and significant increases in CVP and SVI. Furthermore, the dynamic lung compliance decreased significantly after prone positioning (Table 2). In addition, no significant change in cardiac index was observed between the prone and supine positions. With volume expansion, MAP, CVP, SVI, and cardiac index significantly increased, whereas HR, IJVV, SVV, PPV, and dynamic lung compliance significantly decreased (Table 2).

**Table 2:** Changes in hemodynamic and respiratory variables among all patients

Variables	T1	T2	T3
HR (beat·min^-1^)	66.50 ± 8.36	63.71 ± 8.88^a^	60.05 ± 8.28^b^
MAP (mm Hg)	80.36 ± 10.40	76.96 ± 8.14^a^	79.60 ± 6.55^b^
CVP (cm H_2_O)	3.42 ± 1.35	4.73 ± 2.59^a^	8.17 ± 2.53^b^
IJVV (%)	38.09 [20.94-49.09]	24.75 [14.37-33.76]^a^	7.75 [3.13-10.99]^b^
SVV ( %)	14.52 ± 4.01	12.84 ± 4.08^a^	8.69 ± 3.53^b^
PPV (%)	12.18 ± 3.52	11.27 ± 2.58^a^	7.67 ± 2.23^b^
Cardiac index (l·min^-1^·m^-2^)	2.35 ± 0.33	2.34 ± 0.36	2.64 ± 0.39^b^
SVI (ml·m^-2^)	35.67 ± 5.68	37.18 ± 6.32^a^	44.41 ± 6.67^b^
C_dynamic_ (ml·(cm H_2_O)^-1^)	31.57 ± 6.54	28.61 ± 5.66^a^	27.17 ± 5.03^b^

Date are presented as mean ± standard deviation or median [interquartile range]

*HR* hear rate, *MAP* inveasive mean arterial pressure, *CVP* central venous pressure, *IJVV* respiratory variation in the internal jugular vein, *SVV* stroke volume variation, *PPV* pulse pressure variation, *SVI* stroke volume index, *Cdynamic* dynamic lung compliance

^a^Denotes a significant difference between T2 and T1

^b^Denotes a significant difference between T3 and T2

### Changes in hemodynamic and respiratory parameters after volume expansion between responders and non-responders

**Fig. 4:**
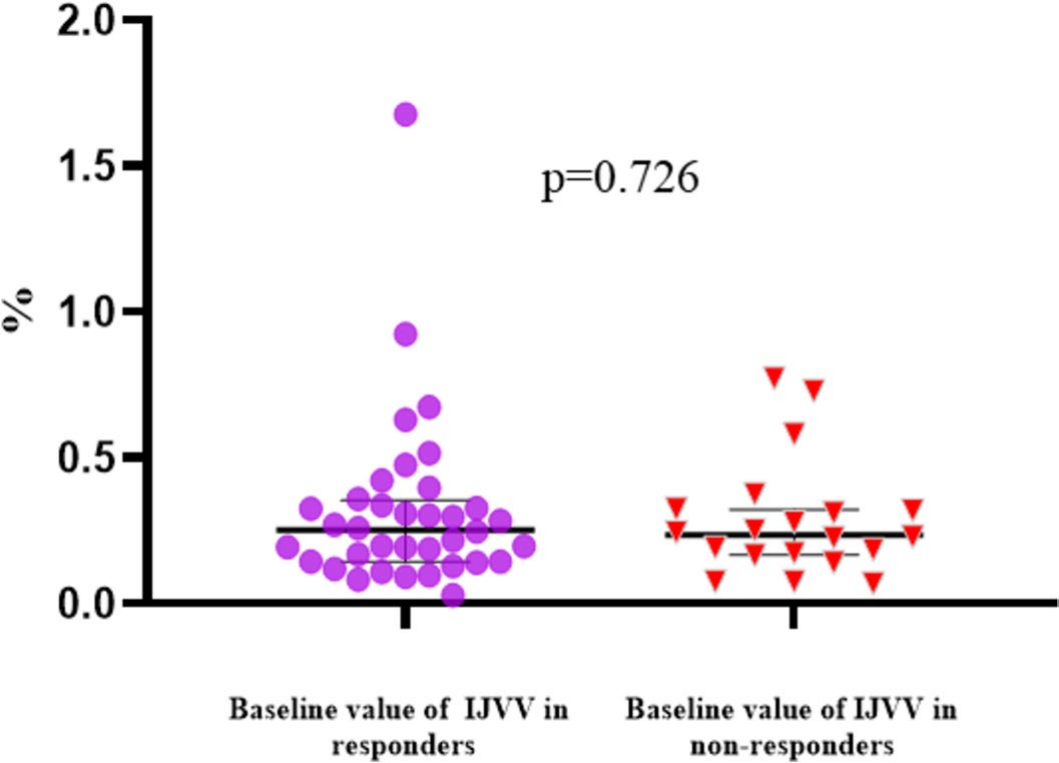
Baseline values of IJVV at T2 in responders and non-responders. IJVV, respiratory variation in the internal jugular vein

Baseline HR, CVP, SVV, PPV, and dynamic lung compliance did not significantly differ between the responders and non-responders before volume expansion, whereas MAP, cardiac index, and SVI were significantly lower in the responders (Table 3, Fig. 2). Furthermore, no significant difference in baseline IJVV was found between responders and non-responders (Table 3, Fig. 4). In both responders and non-responders, volume expansion significantly increased CVP and SVI, and significantly decreased HR, IJVV, SVV, PPV, and dynamic lung compliance, whereas MAP and cardiac index increased significantly only in responders after volume expansion (Table 3). After 7 ml·kg^-1^ fluid loading, greater absolute increases (△) in MAP, SVI, and cardiac index were observed in responders than in non-responders (Supplementary Table S1, Fig. 2).

**Table 3:** Effects of volume expansion on hemodynamic and respiratory parameters in responders and non-responders

Variables	Responders (*n*=36)		Non-responders (*n*=20)	
	T2	T3	T2	T3
HR (beat·min^-1^)	63.94 ± 8.71	60.28 ± 8.37^a^	63.30 ± 9.38	59.65 ± 8.31^a^
MAP (mm Hg)	75.03 ± 7.31	79.55 ± 6.68^a^	80.06 ± 8.64^b^	79.67 ± 6.52
CVP (cm H_2_O)	4.79 ± 1.93	8.48 ± 2.14^a^	4.63 ± 2.31	7.63 ± 3.07^a^
IJVV (%)	25.89 [14.35-35.89]	4.63 [3.13-10.99]^a^	23.66 [16.73-32.27]	7.91 [3.05-10.65]^a^
SVV (%)	12.61 ± 3.46	8.15 ± 2.96^a^	13.26 ± 5.08	9.39 ± 4.35^a^
PPV (%)	11.50 ± 2.72	7.56 ± 2.41^a^	10.85 ± 2.30	7.85 ± 1.92^a^
Cardiac index (l·min^-1^·m^-2^)	2.23 ± 0.30	2.66 ± 0.41^a^	2.53 ± 0.38^b^	2.61 ± 0.36
SVI (ml·m^-2^)	35.40 ± 5.93	44.55 ± 6.87^a^	40.40 ± 5.83^b^	44.17 ± 6.47^a^
Cdynamic (ml·(cm H_2_O)^-1^)	29.35 ± 3.11	28.18 ± 2.99^a^	27.41 ± 4.57	25.53 ± 3.20^a^

Date are presented as mean ± standard deviation or median [interquartile range]

*HR* hear rate, *MAP* mean arterial pressure, *CVP* central venous pressure, *IJVV* respiratory variation in the internal jugular vein, *SVV* stroke volume variation, *PPV* pulse pressure variation, *SVI* stroke volume index, *Cdynamic* dynamic lung compliance

^a^Denotes a significant difference between T3 and T2

^b^Denotes a significant difference between responders and non-responders at the same timepoint

Neither the baseline IJVV in the prone position nor the percentage change in IJVV after fluid loading correlated with the percentage change in SVI after volume expansion (Supplementary Fig. S1, Fig. 2).

### Performance of IJVV, PPV, SVV, and the combination of IJVV and PPV in predicting fluid responsiveness

**Fig. 5:**
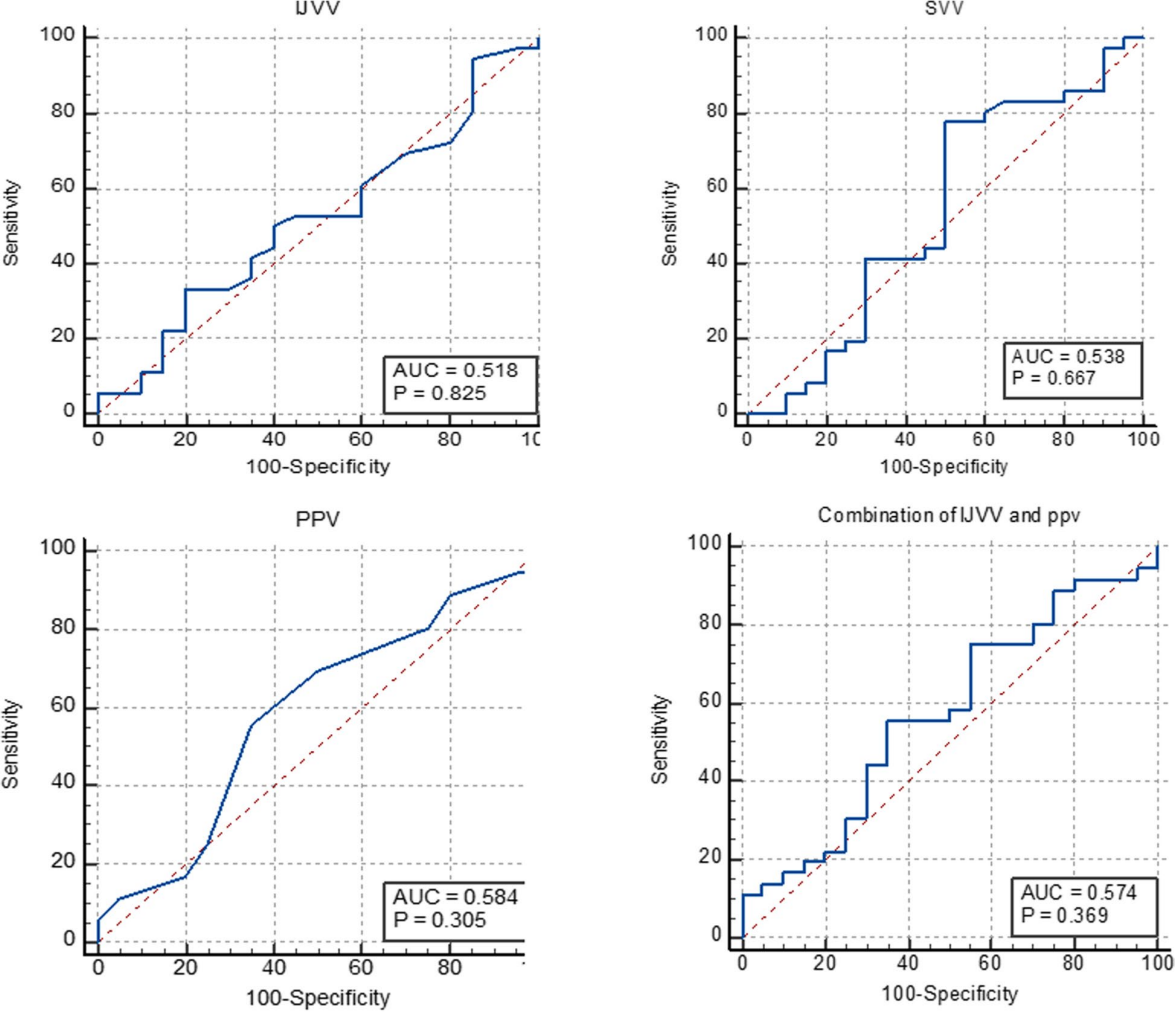
ROC curves for the baseline values of the parameters used for predicting fluid responsiveness. IJVV, respiratory variation in the internal jugular vein; SVV, stroke volume variation; PPV, pulse pressure variation

The diagnostic performance of baseline IJVV in the prone position is shown in Table 4. The AUC of IJVV was 0.52 (95% CI, 0.38–0.65; Fig. 5), and the best threshold was 0.32, corresponding to a sensitivity of 33.33% and a specificity of 80.00%. The utility of the baseline values of PPV, SVV, and the combination of IJVV and PPV for indexing fluid responsiveness are indicated in Table 4. None of these parameters could be considered an accurate diagnostic test (Fig. 5).

**Table 4:** Diagnostic performance of the baseline values of the hemodynamic variables in predicting fluid responsiveness

Variables	AUC (95% CI)	Optimal Cut-off value (%)	Sensitivity (%)	Specificity (%)	Positive likelihood ratio	Negative likelihood ratio	Youden index	*P* value
IJVV	0.52 (0.38-0.65)	32.00	33.33	80.00	1.67	0.83	0.13	0.83
SVV	0.54 (0.40-0.67)	14.30	77.78	50.00	1.56	0.44	0.28	0.67
PPV	0.58 (0.45-0.71)	11.00	55.56	65.00	1.59	0.68	0.21	0.31
Combination of IJVV and PPV	0.57 (0.43-0.71)	64.00	55.56	65.00	1.59	0.68	0.21	0.37

*IJVV* respiratory variation in the internal jugular vein, *SVV* stroke volume variation, *PPV* pulse pressure variation

## Discussion

To our knowledge, this is the first study evaluating the diagnostic performance of IJVV in predicting fluid responsiveness in patients with AIS undergoing PSF surgeries with low Vt ventilation. The main finding of our study was that baseline IJVV was not a good predictor of fluid responsiveness in patients in the prone position under protective ventilation. Second, our findings indicated that the baseline value of PPV and SVV did not predict fluid responsiveness when a lung-protective ventilatory strategy and prone position were applied; moreover, the combination of IJVV and PPV was unreliable in predicting fluid responsiveness in this surgical setting.

AIS is a form of lateral curvature of the spine, which occurs in the absence of an explicit medical cause [31]. Adolescents found to have scoliosis of at least 40° typically undergo surgical correction with PSF to prevent neurologic deficits, further deformation, cardiopulmonary compromise, and resultant pain [32]. PSF surgery for the treatment of AIS has been reported to be associated with substantial perioperative bleeding, which often leads to massive blood transfusion and fluid administration [33]. Furthermore, adolescent patients have different fluid requirements from those of adults, for whom inappropriate use of intravenous fluids may lead to more serious consequences [34]. Therefore, predictors of fluid responsiveness or intravascular volume monitoring are greatly needed for hemodynamic optimization in adolescent patients.

Previous studies have demonstrated that prone position induces a lot of physiologic changes in the respiratory and cardiovascular system. Specifically, it may decrease cardiac index and the compliance of the respiratory system; and increase CVP, PPV, and SVV [35–37]. In agreement with previous findings, the prone position increased CVP in our study. However, we did not observe a decrease in cardiac index, and an increase in PPV and SVV. This discrepancy among studies might be due to differences in the level of cardiac preload and stroke volume. Typically, a higher intra-abdominal pressure (IAP) after prone positioning might collapse the inferior vena cava and lead to a decrease in the venous return and cardiac preload [38]. In the present study, the six pads allowed the abdomen to hang completely freely, and prevented abdominal compression and an influence of the abdominal viscera on the movement of the diaphragm during ventilation. Therefore, we speculate that the equipment used for prone positioning might have minimized the increase in IAP while having minimal effects on cardiac preload. Furthermore, continuous fluid administration during the study period might also have contributed to the minimal change in cardiac index. In addition, continuous fluid supplementation and increased cardiac preload after prone positioning [39] might ultimately lead to decreases in PPV and SVV.

Over the past several decades, enormous efforts have been made to discover non-invasive measures and alternatives to assess volume status. IJVV, a noninvasive and easily determined predictor, has recently been confirmed to accurately assess fluid responsiveness in multiple clinical settings [19–21, 40]. Nonetheless, our study did not demonstrate that baseline IJVV can be used to discriminate between responders and non-responders to fluid expansion among patients with low Vt ventilation in prone position. Notably, although the studies aforementioned showed a desirable predictive effect of IJVV on fluid responsiveness in various clinical settings, they did not include patients with low Vt ventilation under prone position who had different hemodynamic characteristics. We believe that the undesirable predictive effect of IJVV in our study might be explained by the following reasons. First, Vt < 8 ml·kg^-1^ was used in this study, thus leading to small variations in intrathoracic pressure and preload [41]. Therefore, the IJVV in response to ventilation were theoretically expected to be small, regardless of volume status. Furthermore, prone position altered both CVP and the effective circulating blood volume, owing to the impeded venous return resulting from decreased respiratory compliance and increased intrathoracic pressure [42] , which would decrease IJV distensibility; consequently, this predictor might have yielded false negative results.

Conflicting results have been found regarding the ability of PPV and SVV to predict fluid responsiveness in the prone position and under protective ventilation [26, 35, 36, 43–45]. The present study indicated that the baseline values of PPV and SVV could not be used to predict fluid responsiveness in conditions of low Vt ventilation in the prone position. This finding was not unexpected, given that the predictive value of PPV and SVV depends on not only ventilatory settings but also transmission of airway pressure to the intrathoracic structures [45–47]. This transmission is negatively associated with respiratory system resistance [46]. Therefore, under the conditions of low respiratory system compliance and low Vt ventilation in our study, the effect of stroke volume change caused by ventilation was weakened; thus, a high false negative rate was expected. Furthermore, in contrast to a previous study [19], we did not find that the combination of IJVV and PPV increased the sensitivity and specificity of fluid responsiveness prediction. This difference between studies reflects the complicated nature of heart-lung interactions, and implies that the results should be interpreted with caution, particularly under different clinical circumstances.

A 6% HES 130/0.4 infusion was selected to maintain a sustained plasma volume expansion equal to the amount administered [48]. While it is well-known that HES has been suspended from the European market due to adverse effects, including renal dysfunction, increased mortality, and an increased incidence of coagulopathies, in critically ill patients, especially patients with sepsis [49–51]. The safety and effectiveness of HES is likely to differ when used in surgical patients rather than critically ill ones. Recent studies have demonstrated that 6% HES 130/0.4 is not associated with renal dysfunction and increased mortality in patients undergoing surgical procedures [52–59]. In addition, the combination of HES with crystalloids has been shown to have clear advantages, including fewer complications, a higher rate of disability-free survival, and a shorter length of stay compared to crystalloid treatment only [52, 60, 61]. In conclusion, HES 130/0.4 was shown to be safe and efficacious in the perioperative setting when used with appropriate indications. According to a previously published study, children > 12 years of age have much greater reading comprehension and problem-solving ability than younger children [62]. This ability may help to increase an understanding of the study and compliance during the trial. Therefore, we restricted patients to 12–18 years of age.

Collectively, our findings suggested that the development of more specific indicators is warranted. Interestingly, recent studies have demonstrated that tidal volume challenge (Vt adjustment from 6 to 8 ml·kg^-1^) can compensate for the limitation of PPV under low Vt ventilation [26, 63, 64]. However, whether this method can be applied in prone position remains controversial and requires further investigation.

The present study has several limitations. First, all measurements were performed by the same operator, technical errors might have existed, because even slight pressure might have caused significant changes in IJV diameter and cross-sectional images during the measurement of IJVV. However, intra-observer variability was not calculated in this study. Second, because we did not measure IAP in this study, we could not estimate the effect of IAP on the predictive values of target hemodynamic parameters in the prone position. Third, the single-center nature of the present study may limit the generalizability of the findings. Fourth, we conducted this study on a limited number of patients, and type II error might have been present; thus, additional studies with larger sample sizes might be necessary to identify statistical differences. Fifth, our results were based on patients in the prone position, in which six pads were used to allow the abdomen to hang completely freely. Because other positions can cause different alterations in hemodynamic and respiratory mechanics, the results might differ for various types of operating frames or tables used. Sixth, although validation of the FloTrac/Vigileo device in measuring CO has been confirmed by numerous studies, the accuracy of this uncalibrated system is still a matter of debate involving patients with severe aortic valve regurgitation, severe arrhythmias and abnormal left ventricular function, using high-dose vasopressor as well as undergoing laparoscopic procedures [8, 65]. Finally, because our results were acquired from serial measurements, errors in the precision of the system might have influenced the reliability of the measurements.

## Conclusions

In conclusion, this study was not able to demonstrate that IJVV is a valuable indicator of fluid responsiveness after a volume expansion of 7 ml·kg^-1^ colloid in patients with AIS undergoing PSF during low Vt ventilation. Moreover, the baseline values of PPV, SVV, and the combination of IJVV and PPV did not predict fluid responsiveness in this surgical setting.

### Supplementary Information

**Additional file 1: Supplementary Table S1.** Comparison of absolute changes in hemodynamic parameters after volume expansion between responders and non-responders.

**Additional file 2: Supplementary Fig. S1.** Correlation of baseline IJVV and the percentage change in IJVV after fluid loading with an increase in SVI after volume expansion.

## Data Availability

All data supporting the findings of this study are available within the paper and its Supplementary Information.
