# Supplementary material for: Respiratory variation in the internal jugular vein does not predict fluid responsiveness in the prone position during adolescent idiopathic scoliosis surgery: a prospective cohort study
Source: BMC Anesthesiol. 2023 Nov 6;23:360. doi: 10.1186/s12871-023-02313-8 (PMC10626766; doi:10.1186/s12871-023-02313-8)
Supplement: Supplementary file 1 — Additional file 1: Supplementary Table S1. Comparison of absolute changes in hemodynamic parameters after volume expansion between responders and non-responders. [file 12871_2023_2313_MOESM1_ESM.docx]

Supplementary Table S1 Comparison of absolute changes in hemodynamic parameters after volume expansion between responders and non-responders.

| Variables | Responders | Non-responders | p value |
| --- | --- | --- | --- |
| △HR (beat·min^-1^) | -3.67 ± 4.99 | -3.65 ± 5.82 | 0.99 |
| △MAP (mm Hg) | 4.52 ± 7.54 | -0.39 ± 8.76 | 0.04 |
| △CVP (cm H_2_O) | 3 [3-4] | 3 [2-4] | 0.32 |
| △IJVV (%) | -18.50 [-28.00-(-10.00)] | -17.50 [-28.00-(-7.50)] | 0.41 |
| △SVV ( %) | -4.40 ± 4.26 | -3.80 ± 3.64 | 0.21 |
| △PPV (%) | -3.61 ± 2.21 | -1.64 ± 0.48 | 0.16 |
| △Cardiac index (l·min^-1^·m^-2^) | 0.36 [0.19-0.66] | 0.16 [-0.19-0.28] | <0.001 |
| △SVI (ml·m^-2^) | 8 [7-11] | 4 [2-5] | <0.001 |

Date are presented as mean ± standard deviation or median [interquartile range].

HR, hear rate; MAP, inveasive mean arterial pressure; CVP, central venous pressure; IJVV, respiratory variation in the internal jugular vein; SVV, stroke volume variation; PPV, pulse pressure variation; SVI, stroke volume index.
