# Supplementary figures and images for: Respiratory variation in the internal jugular vein does not predict fluid responsiveness in the prone position during adolescent idiopathic scoliosis surgery: a prospective cohort study
Source: BMC Anesthesiol. 2023 Nov 6;23:360. doi: 10.1186/s12871-023-02313-8 (PMC10626766; doi:10.1186/s12871-023-02313-8)

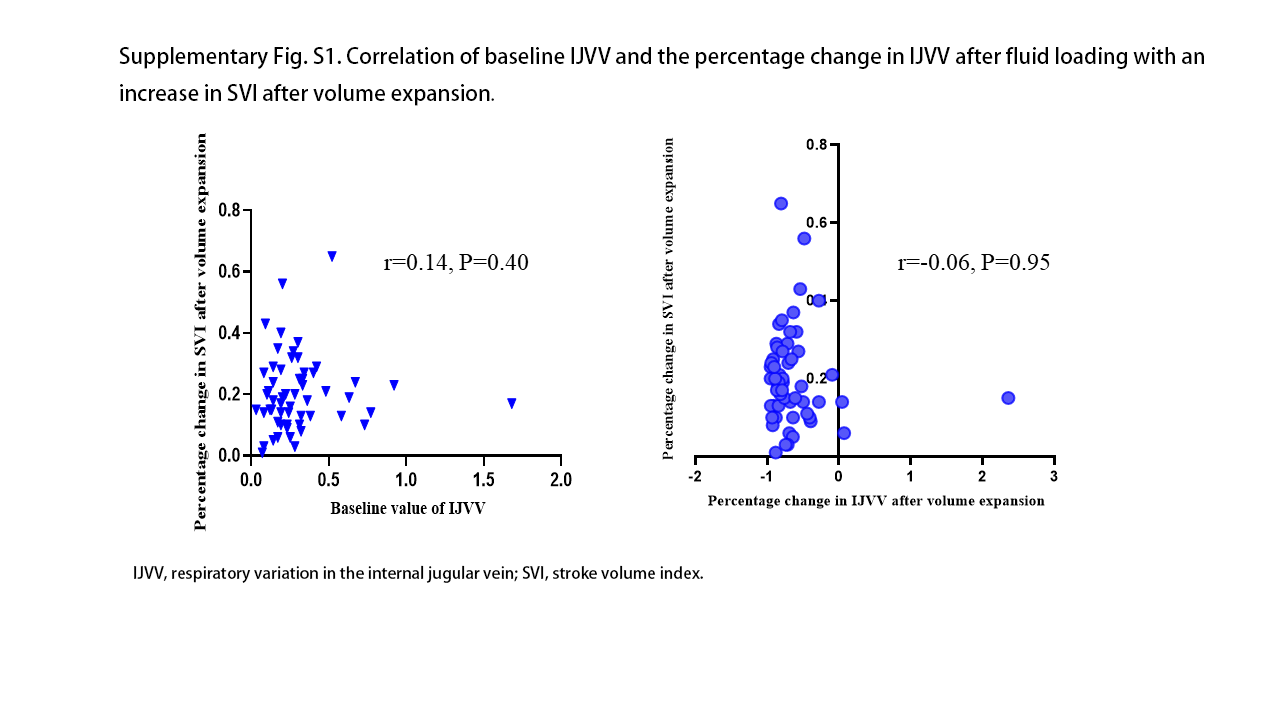

Supplement: Supplementary file 2 — Additional file 2: Supplementary Fig. S1. Correlation of baseline IJVV and the percentage change in IJVV after fluid loading with an increase in SVI after volume expansion. [file 12871_2023_2313_MOESM2_ESM.tif]
